# Hypertension associated with systemic therapy for hepatocellular carcinoma and management strategies

**DOI:** 10.3389/fphar.2026.1780200

**Published:** 2026-05-14

**Authors:** Yuqing Li, Boxuan Wang, Dan Zhang, Ying Xin, Xingyu Wang

**Affiliations:** 1 The People’s Hospital of Qionghai City, Qionghai, China; 2 China-Japan Union Hospital of Jilin University, Changchun, China; 3 The First Hospital of Jilin University, Changchun, China; 4 Huancheng Township Health Center Hospital, Yushu, China; 5 Shenyang Institute of Engineering, Shenyang, China

**Keywords:** antiangiogenic therapy, bevacizumab, cancer therapy-related hypertension, hepatocellular carcinoma, immune checkpoint inhibitors, immunotherapy, tyrosine kinase inhibitors

## Abstract

Antiangiogenic therapy and immune checkpoint inhibitors represent two cornerstone approaches in the current systemic treatment of patients with hepatocellular carcinoma (HCC). Since the advent of the immunotherapy era, the survival of patients with HCC, particularly those with unresectable disease, has improved significantly. However, both drug classes carry the potential for vascular injury, with hypertension being a notable adverse event. Antiangiogenic agents, such as lenvatinib and bevacizumab, frequently induce hypertension. Given that the main systemic regimens for HCC often combine multiple agents prone to cause hypertension, effective management of blood pressure is crucial for long-term adherence to cancer treatment and survival. However, patients with HCC often present with underlying conditions such as cirrhosis, portal hypertension, and ascites. These conditions themselves or their treatments, such as diuretics and non-selective beta-blockers, may lower blood pressure, which is often accompanied by hypoperfusion of vital abdominal organs, particularly the kidneys, posing greater harm than hypertension alone. Consequently, blood pressure management in patients undergoing systemic HCC therapy is highly complex, necessitating the involvement of a ultidisciplinary team collaboration in future practice.

## 1 Introduction

Primary liver cancer has become the sixth most common cancer globally and the third leading cause of cancer-related mortality (Rumgay et al., 2022; Bray et al., 2024; Chan et al., 2025). According to estimates from GLOBOCAN 2022, the annual number of new cases of liver cancer worldwide is projected to increase from 865,000 in 2022 to approximately 1.5–1.6 million by 2050, positioning liver cancer as a substantial global economic burden (Bray et al., 2024; Chan et al., 2025). Primary liver cancer encompasses three main pathological types: hepatocellular carcinoma (HCC), intrahepatic cholangiocarcinoma, and combined hepatocellular-cholangiocarcinoma, with HCC accounting for 85%–90% of cases. The systemic treatment of HCC has undergone a significant transformation with the advent of the immunotherapy era. Current first-line clinical regimens include primarily small-molecule antiangiogenic agents, macromolecular antiangiogenic agents, immune checkpoint inhibitors (ICIs), and their combinations (Vogel et al., 2022). Zhou et al., (2025) traditional chemotherapeutic agents, such as platinum-based compounds and fluoropyrimidines, are now more commonly used in transarterial chemoembolization (TACE) and are less frequently employed as first-line systemic therapies. Hypertension is a common adverse effect associated with small-molecule antiangiogenic agents, macromolecular antiangiogenic agents, and ICIs (Table 1). In particular, patients with HCC may also present with underlying conditions such as portal hypertension and ascites. Systemic therapy-induced hypertension management strategies differ in many aspects from those of other types of cancer and thus, pose significant clinical challenges. Conducting related clinical research is also particularly difficult. Therefore, the aim of this comprehensive review of the literature is to address these clinical concerns.

**TABLE 1:** Hypertension associated with systemic therapy for HCC in clinical studies.

First-line systemic therapies	Pharmaceutical preparations	Incidence of hypertension	Types of HCC	Literature serial number
Small-molecule antiangiogenic agents	*Lenvatinib*	A higher incidence of all-grade hypertension compared with sorafenib (approximately 2.65 times greater)	First-line treatment of advanced HCC	Luo et al. (2022), Xiaoyuan Chu et al. (2025)
		The incidence of treatment-emergent hypertension of any grade was 42%	First-line treatment of unresectable HCC	Kudo et al. (2018)
	*Sorafenib*	Hypertension was the predominant manifestation among adverse events of grade ≥3	——	Zhou J et al. (2025)
	*Apatinib*	The incidence of treatment-emergent hypertension of any grade was 48.3%	Second-line or later treatment of advanced HCC	Qin et al. (2021)
Small-molecule antiangiogenic monotherapy combined with ICIs	*Lenvatinib plus Pembrolizumab*	Hypertension was the most common of these adverse events (57 [24%] vs. 18 [7%])	TACE combined with lenvatinib plus pembrolizumab for patients with unresectable, non-metastatic HCC	Xiaoyuan Chu et al. (2025), Kudo et al. (2025)
	*Camrelizumab plus Apatinib*	The incidence of hypertension was 38%, compared with 15% in the control group	First-line treatment of unresectable HCC	Qin et al. (2023)
Macromolecular antiangiogenic agents combined with ICIs	*Atezolizumab plus Bevacizumab*	The incidence of grade 3 hypertension was 12%	Unresectable	Cheng et al. (2022)
		The most common grade 3 or 4 event was hypertension (15.2%)		Finn et al. (2020)
	*Toripalimab plus Bevacizumab*	The most common grade 3–4 adverse event was hypertension (26 [16%] vs. 19 [12%])	Unresectable or metastatic	Shi et al. (2025)
		The incidence of grade 1–2 hypertension was 22%		
ICI or dual ICI combination	*the STRIDE Regimen (Durvalumab plus Tremelimumab)*	The incidence of grade 3 hypertension remained below 5% for both the combination and monotherapy arms	Unresectable	Abou-Alfa et al. (2022)
Traditional chemotherapeutic agents	*the FOLFOX Regimen (Oxaliplatin plus Fluorouracil)*	No grade 3 or 4 hypertension adverse events were observed in this study	Patients who had locally advanced or metastatic HCC and were ineligible for curative resection or local treatment	Qin et al. (2013)

## 2 First-line systemic therapies for HCC and associated hypertension

### 2.1 Small-molecule antiangiogenic agents

Small-molecule tyrosine kinase inhibitors (TKIs) constitute a vital component of systemic therapy for HCC. Their mechanisms of action primarily involve antiangiogenic effects achieved by inhibiting multiple targets, including vascular endothelial growth factor (VEGF) receptors (VEGFR) (Li et al., 2024). However, cancer therapy-related hypertension (CTR-HTN) is among the most common and dose-dependent adverse events associated with these agents. The time to onset of CTR-HTN varies depending on the specific antitumor drug administered (Society of Integrative Cardio-Oncology C.A.-C.A.Expert Panel on Chinese Expert Consensus on Integrated Management of Cance r-Related Hypertension, 2024). As summarized in Table 1, TKIs such as lenvatinib, sorafenib, and apatinib are linked to distinct rates of CTR-HTN. A systematic review and meta-analysis focusing on lenvatinib demonstrated a higher incidence of all-grade hypertension compared with sorafenib, with a risk approximately 2.65 times greater (HR 2.65, 95% CI 1.78–3.93, P < 0.00001) (Luo et al., 2022; Xiaoyuan Chu et al., 2025). The incidence of treatment-emergent hypertension of any grade was 42% with lenvatinib in a first-line treatment trial and 48.3% with apatinib in a later-line (second-line or beyond) treatment trial (Kudo et al., 2018; Qin et al., 2021). This form of drug-induced hypertension typically emerges early in the treatment course, particularly within the 2–6 weeks following therapy initiation, and its severity shows a close correlation with drug dosage. The rapid and early onset of elevated blood pressure presents a significant challenge for out-of-hospital management.

### 2.2 Small-molecule antiangiogenic monotherapy combined with ICIs

The combination of small-molecule TKIs with ICIs is associated with a further elevated incidence of hypertension. Moreover, we consider that hypertension induced by this combination therapy may also be the most severe. A systematic review on the safety of TACE/hepatic arterial infusion chemotherapy (HAIC) combined with TKIs and ICIs for unresectable HCC indicated that among all-grade adverse events, hypertension was among the top three and was the most common severe adverse event (Ke et al., 2022). In clinical studies, the incidence of grade 3 hypertension with TKI + ICI combinations can reach 15%–25%. Specifically, lenvatinib plus pembrolizumab combined with TACE yielded a hypertension incidence of 24% (vs. 7% in controls) (Xiaoyuan Chu et al., 2025; Kudo et al., 2025). Additionally, camrelizumab plus apatinib was associated with a 38% incidence (vs. 15% in controls) (Qin et al., 2023). The underlying mechanisms of hypertension in this combined regimen are more complex and involve not only the direct inhibition of the VEGF pathway by TKIs but also may be compounded by immune-related adverse effects. ICIs may contribute to elevated blood pressure by inducing immune-mediated vasculitis, leading to altered vascular permeability and endothelial dysfunction. Additionally, ICIs can cause immune-related nephritis, which damages nephrons, and leads to sodium retention and over-activation of the renin-angiotensin-aldosterone system (RAAS), both potent drivers of hypertension. This immunologically-mediated rise in blood pressure may differ in its time of onset from the toxicity profile of TKIs alone. Thus, blood pressure fluctuations can be more volatile and less predictable, presenting new and significant challenges for clinical management.

### 2.3 Combination of macromolecular antiangiogenic agents and ICIs

Macromolecular antiangiogenic agents, such as bevacizumab, are not typically used as monotherapy in the systemic treatment of HCC but are combined with ICIs. This combination regimen also induces hypertension. In the landmark IMbrave150 study (Cheng et al., 2022), the incidence of grade 3 hypertension in the atezolizumab plus bevacizumab group was 12%, which was higher than the 9% observed in the sorafenib monotherapy group. The most common grade 3 or four event with atezolizumab–bevacizumab was hypertension (15.2%) (Finn et al., 2020). A randomized, open-lable phase 3 trial evaluating Toripalimab plus bevacizumab for patients with unresectable or metastatic HCC reported that the most common (incidence ≥5% in the toripalimab plus bevacizumab group) grade 3–4 adverse events were hypertension (26 [16%] in the toripalimab plus bevacizumab group vs. 19 [12%] in the sorafenib group) (Shi et al., 2025). Compared with hypertension induced by small-molecule TKIs, hypertension caused by macromolecular agents typically manifests slightly later, with the peak blood pressure elevation potentially delayed until weeks three–4 after treatment initiation. This delayed onset can lead patients to become less vigilant about blood pressure monitoring during the critical early out-of-hospital period. Furthermore, the direct vascular effects of bevacizumab, an anti-VEGF antibody, combined with the immunomodulatory effects of ICIs, result in a more complex pathophysiology of blood pressure changes.

### 2.4 ICI or dual ICI combination

The incidence of ICI-induced hypertension or dual ICI combinations is significantly lower than that associated with antiangiogenic agents (Minegishi et al., 2022). Taking the STRIDE regimen (durvalumab plus tremelimumab) as an example, the incidence of grade 3 hypertension remained below 5% for both the combination and monotherapy arms, which is substantially lower than that observed in the sorafenib control group (Abou-Alfa et al., 2022). The exact mechanism of ICI-related hypertension has not been fully elucidated but may be indirectly linked to immune-mediated phenomena. Preclinical studies have demonstrated that ICIs induce endothelial dysfunction via ICAM-1 and iNOS-mediated leukocyte infiltration and inflammation, and upregulate the expression of VCAM-1, ICAM-1 and other adhesion molecules on endothelial cells to further promote leukocyte adhesion, infiltration and vascular inflammation (Efentakis et al., 2024; Poels et al., 2020). As the chronic immune activation caused by ICIs to exert antitumor effects can disrupt vascular homeostasis, ICIs may elevate blood pressure through immune-related inflammation and vascular damage (Watson et al., 2025). Although its onset is often insidious, and the magnitude of blood pressure elevation is generally milder, vigilance is still warranted. As ICIs do not directly target the VEGF pathway, they have a lesser impact on both essential hypertension and portal hypertension, which is an important advantage in specific patient populations.

### 2.5 Regimens based on traditional chemotherapeutic agents

Traditional chemotherapy, represented by the FOLFOX regimen (oxaliplatin plus fluorouracil), (Qin et al., 2013) remains a first-line treatment option in the latest clinical guidelines for patients with locally advanced and metastatic HCC that is ineligible for surgery or locoregional therapy (Li et al., 2022). In contrast to targeted and immunotherapy, hypertension is not a primary adverse effect of traditional chemotherapeutic agents, and has an incidence of grade 3 hypertension typically below 3%. However, certain chemotherapeutic drugs, such as oxaliplatin, may exert cumulative toxicity on the vascular system and kidneys with long-term use, potentially leading to persistent hypertension. Oxaliplatin, a classical platinum-based chemotherapeutic agent, can induce peripheral neuropathy (oxaliplatin-induced peripheral neuropathy, OIPN), which exerts vascular toxicity by impairing the endothelial glycocalyx in peripheral capillaries and consequently elevating vascular permeability (Kuroda et al., 2024). The onset of this form of hypertension is usually insidious, often becoming apparent only after the cumulative administration of four–five cycles.

## 3 Factors influencing blood pressure in patients with hepatocellular carcinoma

**FIGURE 1:**
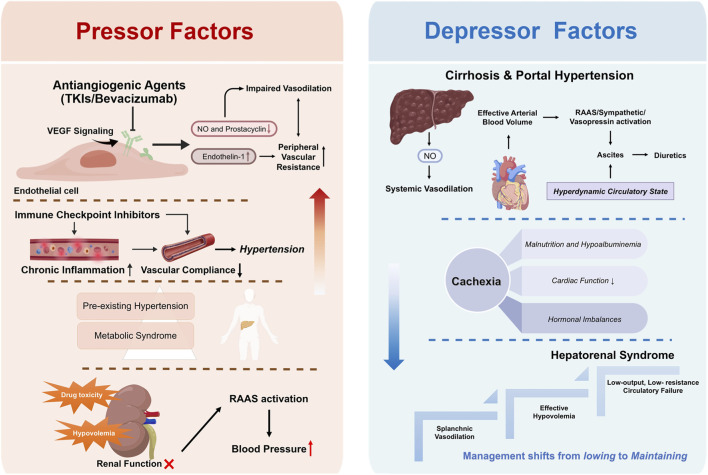
Pressor vs. depressor factors in HCC with cirrhosis and portal hypertension. Abbreviations: TKI, tyrosine kinase inhibitors; VEGR, vascular endothelial growth factor; RAAS, renin-angiotensin-aldosterone system; NO, nitric oxide.

The management of hypertension in patients with HCC represents a profoundly complex clinical challenge, based on the dynamic interplay of multiple, often opposing, physiological forces (Figure 1). This complexity arises from the simultaneous presence of potent and depressor influences within the patient, creating a fragile and precarious equilibrium. The introduction of systemic therapies, particularly antiangiogenic agents, critically disrupts this balance, making the concept of “bidirectional regulation” paramount. These therapeutic agents directly induce increased blood pressure through specific pharmacological mechanisms. However, underlying liver cancer and its associated complications, such as portal hypertension, ascites, and hepatorenal syndrome, generate a powerful “depressor” background. This unique pathophysiological state requires clinicians to move beyond conventional hypertension management paradigms. A deep understanding of the specific pathophysiology of each patient is essential to devise a safe and effective individualized blood pressure control strategy. Neglecting either side of this equation can lead to catastrophic outcomes; for example, overly aggressive antihypertensive therapy may precipitate or exacerbate hepatorenal syndrome, whereas inadequately managed drug-induced hypertension may trigger severe cardiovascular or cerebrovascular events. Therefore, accurate identification and careful weighting of these opposing factors constitute the cornerstone of successful management of systemic therapy-related hypertension in HCC.

### 3.1 Pressor factors

Systemic therapy for HCC, especially with antiangiogenic agents, is the most direct and significant driver of blood pressure elevation. The central mechanism involves inhibition of the VEGF signaling pathway. VEGF plays a crucial role in maintaining normal vascular endothelial function and vascular tone by stimulating endothelial cells to produce vasodilatory substances, primarily nitric oxide (NO) and prostacyclin. When antiangiogenic drugs block the VEGF/VEGFR pathway, the endothelial capacity to generate NO is reduced. This leads to impaired vasodilation, a subsequent increase in peripheral vascular resistance, and ultimately a rise in blood pressure. Furthermore, VEGF inhibitors may also contribute to an increase in endothelin-1 levels, a potent vasoconstrictor, thereby amplifying vascular constriction.

Patients with HCC, particularly those whose disease originates from metabolic dysfunction–associated steatotic liver disease (MASLD), often present with a comorbid metabolic syndrome, of which hypertension is a core component. Some patients with HCC may also present with pre-existing essential hypertension. Before starting antitumor therapy, these patients may already exhibit elevated baseline blood pressure or possess a vascular system with pre-existing injury and functional abnormalities. The addition of the pressor effect of antiangiogenic agents makes these individuals more susceptible to sharp, rapid, and difficult-to-control blood pressure surges.

The kidneys are central to long-term regulation of blood pressure, yet their function is highly vulnerable in patients with HCC, especially those with underlying cirrhosis. Effective hypovolemia induced by cirrhosis and reduced renal perfusion can directly injure the renal tubules, among which special attention should be paid to the condition of abdominal hypoperfusion, as it is the most severe type of renal function injury in HCC patients with cirrhosis and can further aggravate renal damage through the synergistic effect of local renal RAAS activation, systemic hypotension and renal hypoperfusion. In addition, antitumor agents and their metabolites can also exert direct nephrotoxic effects, which together lead to the deterioration of renal function. The worsening of renal function can activate the RAAS, leading to increased production of potent vasoconstrictors such as angiotensin II, which subsequently increases blood pressure. At the same time, it should be supplemented that the abnormal activation of RAAS is not only a subsequent result of renal function deterioration, but also can conversely aggravate renal damage itself. Through mechanisms such as constricting renal blood vessels, reducing renal blood flow, and promoting renal interstitial fibrosis, it forms a vicious cycle of “deterioration of renal function - RAAS activation - further renal damage”.

When TKIs are combined with ICIs, the pressor mechanisms become more complex and potentially synergistic. Direct VEGF/VEGFR inhibition by TKIs reduces NO production and elevates endothelin-1 levels. Meanwhile immune-mediated vascular chronic inflammation and endothelial dysfunction induced by ICIs further compromise vascular integrity. Vascular toxic effects, related to accelerated arteriosclerosis from systemic and vascular inflammation induced by ICIs, can lead to complications, sucn as hypertension (Herrmann et al., 2026). Overall, the combination of TKIs and ICIs leads to more complex pressor mechanisms.

### 3.2 Depressor factors

Cirrhosis is the most common pathological background for HCC, with portal hypertension serving as its central complication. In the initial phase of portal hypertension, hepatic fibrosis and regenerative nodules lead to increased intrahepatic vascular resistance and increased vascular tone. Subsequently, vasodilatory substances-most notably nitric oxide-are activated within the splanchnic circulation (Iwakiri and Trebicka, 2021). This results in a progressive vasodilation in both splanchnic and systemic vascular beds, leading to a reduction in the effective volume of arterial blood. This hypovolemic state then activates the neurohumoral systems (including the RAAS, the sympathetic nervous system, and the arginine-vasopressin system), culminating in sodium and water retention and the formation of ascites. Once established, this hyperdynamic circulatory state is characterized by increased cardiac output, tachycardia, decreased systemic vascular resistance, and ultimately a reduction in arterial blood pressure (McGrath and Wentworth, 2024).

In HCC patients receiving antiangiogenic therapy, the opposing hemodynamic forces at the microvascular level create a complex regulatory environment. Portal hypertension triggers the activation of NO, leading to extensive splanchnic vasodilation and a reduction in effective circulating blood volume. The body attempts to maintain effective circulating blood volume by activating the RAAS to generate angiotensin II. Meanwhile, antiangiogenic therapy further suppresses the production of NO in the systemic vasculature. These opposing forces act primarily at the renal afferent arteriole. Here, insufficient renal perfusion resulting from splanchnic vasodilation and vasoconstriction induced by antiangiogenic therapy exert opposite effects on the regulation of the glomerular filtration rate.

Patients with advanced HCC often present with severe cachexia, manifested by progressive weight loss, muscle wasting, fat depletion, and generalized fatigue. Cachexia itself constitutes a significant factor that contributes to hypotension. First, severe malnutrition and hypoalbuminemia lower the plasma oncotic pressure, facilitating the extravasation of intravascular fluid into the interstitial space and therefore exacerbating the deficit in effective circulatory volume. Second, cardiac muscle function can be compromised in patients with cachexia, leading to reduced cardiac output. Third, systemic metabolic dysregulation and hormonal imbalances, such as alterations in cortisol and catecholamine levels, can also affect vascular tone and blood pressure regulation. Consequently, patients with HCC in a cachectic state typically exhibit a low baseline blood pressure and demonstrate an extremely poor tolerance to antihypertensive medications. Even a slight reduction in blood pressure can cause symptoms such as dizziness, fatigue, or even shock.

### 3.3 Impediments to the management of blood pressure in patients with HCC

Hepatorenal syndrome (HRS) is one of the most severe complications in patients with cirrhosis and HCC, representing acute kidney injury (AKI) secondary to liver failure. The pathophysiological hallmark of HRS is severe splanchnic vasodilation and profound effective hypovolemia, which critically impairs renal perfusion. Its hemodynamic profile is characterized by a “low-output, low-resistance circulatory failure,” where, despite a potentially increased cardiac output (hyperdynamic circulation), the marked reduction in systemic vascular resistance results in a low level arterial blood pressure or its gradually decline (Koratala et al., 2024). In this state, any further attempt to reduce blood pressure can act as the “final straw” that overwhelms the patient’s circulatory system, potentially precipitating irreversible renal failure and circulatory collapse. Therefore, for patients at risk for or already diagnosed with HRS, the primary goal of management shifts from “lowering” to “maintaining” blood pressure. The aim of the intervention is to raise mean arterial pressure and promote renal perfusion, which is critical to the overall prognosis of the patient (Simonetto et al., 2020).

## 4 Management strategies for hypertension related to systemic therapy in hepatocellular carcinoma

Currently, the cardio-oncology guidelines of China’s two major academic societies, CACA and CSCO, do not separately address the management of CTR-HTN specific to HCC. Although HCC shares some management strategies for systemic treatment-related hypertension with other malignant tumors, general hypertension management guidelines cannot be simply applied to HCC patients who are potentially complicated with portal hypertension and ascites. Before starting antitumor therapy, CSCO guidelines (Xiaoyuan Chu et al., 2025) recommend that all patients undergo a baseline blood pressure measurement and receive education on healthy lifestyle modifications, and that they should be informed that anti-cancer treatment can induce new-onset hypertension or exacerbate pre-existing hypertension, strongly encouraging them to engage in regular self-monitoring of blood pressure. For HCC patients complicated with portal hypertension and ascites, a multidisciplinary team (MDT) involving hepatology department/digestive department and nephrology department should be convened before treatment to evaluate whether anti-angiogenic therapy may exacerbate portal hypertension and ascites or induce HRS by affecting blood vessels. For patients with cancer and comorbid hypertension, if their blood pressure is already well-controlled, the existing antihypertensive regimen should be maintained. If blood pressure control is suboptimal, treatment should be optimized according to the guidelines to achieve the target blood pressure. Except in cases of grade 3 hypertension, it is generally not recommended to delay the initiation of antitumor therapy solely due to uncontrolled blood pressure.

**FIGURE 2:**
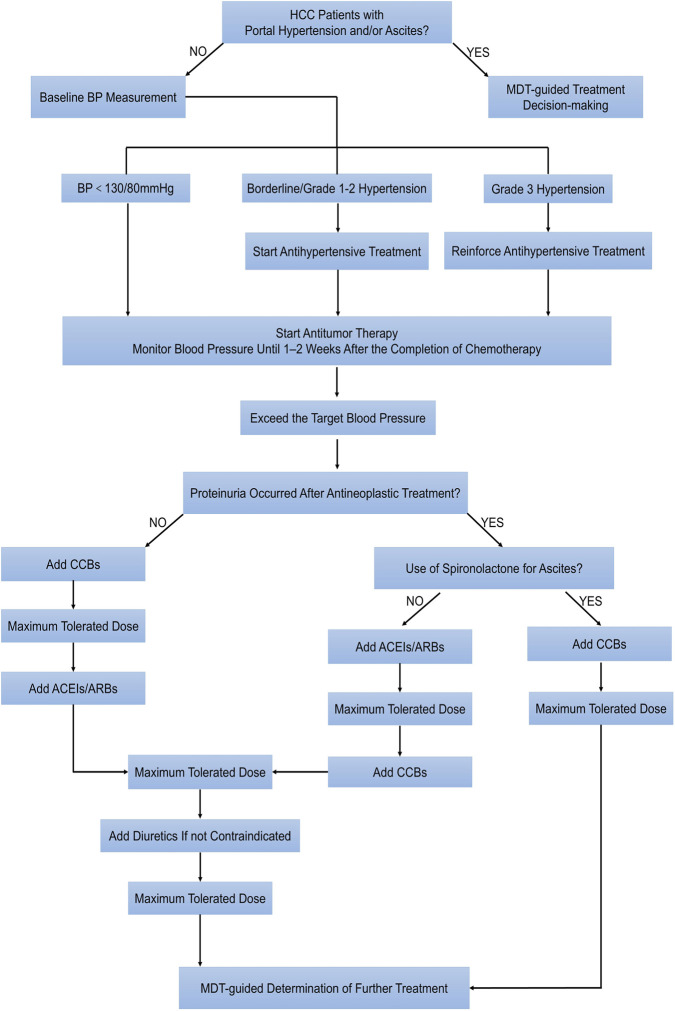
MDT-guided blood pressure management in HCC with portal hypertension and/or ascites. Abbreviations: HCC, hepatocellular carcinoma; BP, blood pressure; MDT, multidisciplinary team; CCB, calcium channel blockers; ACEI, angiotensin-converting enzyme inhibitors; ARB, angiotensin II receptor blockers.

For patients receiving effective antitumor therapy with a favorable prognosis, blood pressure management can often follow general guidelines for the prevention and treatment of hypertension. According to the CSCO guidelines, non-dihydropyridine calcium channel blockers (CCBs) are the preferred choice for CTR-HTN, whereas angiotensin-converting enzyme inhibitors (ACEIs) and angiotensin II receptor blockers (ARBs) are recommended first for patients with proteinuria. However, the threshold for initiating pharmacologic antihypertensive treatment for CTR-HTN and subsequent blood pressure targets must be individualized. This decision should be based on a comprehensive assessment of factors including the patient’s age, estimated life expectancy, the presence of hypertensive damage to the target organ, and other comorbidities. In this review, a flow chart (Figure 2) was developed to systematically summarize the management strategies of hypertension related to systemic therapy in HCC.

### 4.1 Calcium channel blockers

In the out-of-hospital management of systemic therapy-related hypertension in patients with HCC, CCBs are often considered the primary “safe choice” due to their favorable safety profile, tolerability, and convenient dosing regimen. In particular, in contexts where evidence is insufficient and guidelines are not yet definitive, selecting a drug with lower risk and ease of management is crucial to ensuring patient safety. CCBs lower blood pressure by blocking calcium channels in vascular smooth muscle cells, directly dilating peripheral arteries, and thus reduce peripheral vascular resistance. This mechanism of action has minimal overlap with the pathways targeted by antiangiogenic agents, resulting in a relatively low risk of drug-drug interactions and providing a unique advantage in complex polypharmacy scenarios.

### 4.2 Renin-angiotensin-aldosterone system inhibitors

RAAS inhibitors, including ACEIs and ARBs, theoretically offer potential benefits beyond merely lowering blood pressure. They are considered “synergistic” agents that may confer a survival advantage (Barone et al., 2019; Zhang et al., 2022). Their mechanism of action extends beyond RAAS blockade and may involve inhibiting VEGF signaling pathways and exerting antifibrotic effects, potentially creating synergistic effects with antitumor therapy. However, the use of RAAS inhibitors in patients with HCC is a double-edged sword. Their inherent risks to renal function and hemodynamics require rigorous patient selection and close monitoring by clinicians to maximize their benefits within a safe framework.

### 4.3 Diuretics and non-selective beta-blockers

Within the complex clinical landscape of patients with HCC, the use of certain traditional antihypertensive agents is fraught with contradictions and controversy. Diuretics and non-selective beta-blockers (NSBBs) are prime examples. Although these drug classes are integral to standard management in specific contexts, their role in the management of systemic therapy-related hypertension may precipitate obvious side effects. Consequently, for patients who are already being treated with these medications or for whom their use is indicated for specific complications, it is imperative to develop a cautious, individualized, and closely monitored out-of-hospital adjustment strategy. This approach is essential to balance risks and benefits, with the aim of maximizing therapeutic benefit while minimizing potential harm.

Diuretics are a cornerstone of treatment for cirrhotic patients with ascites, although their application is strictly limited when addressing systemic therapy-induced hypertension. Loop diuretics are relatively contraindicated for out-of-hospital management in this setting. The primary risk stems from their potent natriuretic and diuretic effects, which rapidly reduce the effective circulating volume. In patients with cirrhosis, whose hemodynamic state is already precarious, this can easily precipitate prerenal AKI and exacerbate electrolyte imbalances. If clinical necessity mandates their use for severe ascites, they should be administered under rigorous monitoring and typically in combination with an aldosterone receptor antagonist (e.g., spironolactone). Spironolactone, the preferred diuretic for cirrhotic ascites, has a milder mechanism, promoting diuresis by antagonizing the sodium and water retention effects of aldosterone. In the out-of-hospital setting, low-dose spironolactone (25–50 mg/day) may be considered for patients with HCC and concomitant ascites (Chien et al., 2025; Lin et al., 2021). In addition, spironolactone may possess antifibrotic properties, adding a potential ancillary benefit to its use in patients with HCC (Lin et al., 2021). Nevertheless, monitoring of serum potassium and renal function remains mandatory to protect against the risk of hyperkalemia.

NSBBs, such as propranolol and carvedilol, are first-line agents for the primary and secondary prophylaxis of variceal bleeding in cirrhosis. However, their interaction with hypertension management is complex and potentially adverse (Ismail et al., 2025). NSBBs lower portal pressure by blocking β1-receptors, reducing heart rate and cardiac contractility, and β2-receptors, leading to splanchnic vasoconstriction. Crucially, this reduction in cardiac output can impair the compensatory capacity of the body to maintain blood pressure and vital organ perfusion under stress. In patients with advanced cirrhosis, sustained cardiac output is a key defense against profound vasodilation and is critical to preserve renal perfusion. Therefore, NSBB use may mask the true severity of hypertension while simultaneously increasing the risk of hypotension, AKI, and HRS. As previously mentioned, for patients already receiving NSBBs for variceal bleeding prophylaxis, it is necessary to conduct MDT and implement intensive monitoring and the regimen must be quickly reassessed and potentially adjusted should any contraindications to NSBB therapy arise.

## 5 Discussion

The management of hypertension associated with systemic therapy for HCC represents a challenging and rapidly evolving field. Current clinical practice faces significant controversies and deficiencies, the most prominent being the inherent limitations of a single-discipline approach to this complex issue. As discussed in this review, most existing cardio-oncology practice guidelines offer only general recommendations for cancer therapy-related hypertension for all malignancies and often lack dedicated sections that specifically address HCC and expand on the profound impact of concurrent factors, including ascites, portal hypertension, and hepatorenal syndrome.

Simultaneously, a long-standing and contentious hypothesis in oncology indicates that hypertension induced by antiangiogenic agents may be a biomarker of treatment efficacy (Kidoguchi, 2025). It may be speculated that these drugs exert antitumor effects by inhibiting the VEGFVEGFR pathway, with hypertension emerging as a “targeted” adverse event signaling effective pathway blockade (Rini et al., 2011). Hypertension after bevacizumab administration may correlate with favorable prognosis in colorectal cancer (Scartozzi et al., 2009), and treatment-emergent hypertension predicts improved survival in thyroid cancer patients receiving lenvatin (Wirth et al., 2018). A study shows that elevated blood pressure may serve as a favorable prognostic marker, even after adjustment for key variables including age, disease stage, Eastern Cooperative Oncology Group Performance Status (ECOG-PS), and liver reserve function (Shibata et al., 2025). Given that these variables are established determining factor of prognosis of patients receiving lenvatinib, the prognostic value of blood pressure elevation carries important clinical significance (Hiraoka et al., 2019; Yang et al., 2023). Furthermore, hypertension is an early, recognizable adverse event, and since blood pressure monitoring is non-invasive and readily implementable in routine clinical settings, it possesses considerable promise for clinical application (Shibata et al., 2025). However, this view is not without controversy. Critics argue that hypertension itself increases the risk of cardiovascular events and may even necessitate treatment interruption, potentially negating any survival advantage. Therefore, how to scientifically interpret and navigate this controversy is crucial to optimally guide clinical practice.

In some cases, patients do not develop clinical hypertension, but actually exhibit upregulation of vasoactive substances, vascular dysfunction, and insufficient organ perfusion, which are manifestations of decompensation of blood pressure regulation. Obviously, such patients have a shorter survival time. This may explain why patients who develop hypertension might receive a greater survival benefit. Vascular dysfunction induced by systemic therapy for HCC presents a hidden state characterized by normal or low systemic blood pressure yet locally elevated shear stress in the liver. Mechanical forces drive fibrosis via transduction pathways (Mascharak et al., 2024). Hypertension and high shear stress are risk factors for vascular sclerosis and fibrosis in vascular-rich tissues, which can activate relevant pathways to accelerate hepatic fibrosis. Elevated hepatic sinusoidal hydrostatic pressure and abnormal shear stress caused by cirrhosis will further exacerbate this process. Detection of vasoactive substances such as NO/endothelin-1 and angiotensin II can break through the superficial manifestation of blood pressure to reflect the real hemodynamic crisis in the local liver tissue. However, the stratification system in current clinical research on liver diseases remains crude. Studies on HCC-associated hypertension are only able to conduct standardized blood pressure statistics, and the detection of vasoactive substances has not been popularized due to cost, technical thresholds and other factors. This not only hinders the accurate identification of high-risk patients, but also restricts the development of relevant precise diagnosis and treatment strategies.

Furthermore, drugs such as lenvatinib, while inducing hypertension as an adverse effect, may also lead to decreased renal function, reduced urinary sodium excretion, and a significant increase in proteinuria (Saito et al., 2020; Saito et al., 2019; Furuto et al., 2018). While hypertension itself can aggravate renal damage and contribute to proteinuria, it is critical to recognize the renal toxicity of small-molecule antiangiogenic agents. Lenvatinib exerts its nephrotoxic effects through inhibiting VEGF receptors and platelet-derived growth factor receptors (Cavalieri et al., 2018). As VEGF is essential for maintaining glomerular capillary integrity and renal microvascular homeostasis, its blockade by lenvatinib can trigger endothelial injury, renal vasoconstriction and microvascular damage, ultimately leading to proteinuria and renal dysfunction (Cavalieri et al., 2018). Moreover, lenvatinib may lead to thrombotic microangiopathy, a condition marked by microthrombi formation within renal capillaries, which further exacerbates renal impairment (Cavalieri et al., 2018). For these patients, ACEIs or ARBs may be more suitable due to their potential to reduce proteinuria. However, renal function and serum potassium levels require close monitoring. The situation becomes more complex in HCC patients complicated by portal hypertension and ascites. Salt restriction combined with diuretics serves as the fundamental approach for ascites management, yet excessive diuresis may exacerbate renal injury. Spironolactone is established as the diuretic of choice and has been shown to reduce proteinuria, with clinical data supporting this practice (Oiwa et al., 2023; Amini et al., 2025). For patients already receiving spironolactone, CCBs may represent a safer alternative, even in the presence of proteinuria. For newly occurred portal hypertension, ascites, proteinuria or exacerbation of pre-existing conditions induced by anti-angiogenic therapy, MDT involving hepatology department/digestive department and nephrology department is recommended for comprehensive integration of treatment drugs. It is also important to note that clinical trial populations typically exclude patients with severe cardiovascular or renal diseases; therefore, in real-world settings, the incidence of hypertension and subsequent proteinuria may be even higher.

The complexity of systemic therapy-related hypertension in HCC far exceeds the scope of any single medical specialty. It is not merely an antitumor therapy side effect, but a complex pathophysiological problem that affects the cardiovascular, hepatic, and renal systems. Thus, any attempt to address this issue from a single disciplinary perspective risks leading to incomplete or suboptimal management decisions due to inherent limitations from individual viewpoints. The MDT collaboration model, integrating expertise and experience from various fields, offers an optimal framework to navigate this clinical challenge.

An ideal MDT should function as a collaborative, patient-centric platform oriented to problems. Its core membership should at minimum include specialists in oncology, cardiology, hepatology/gastroenterology, nephrology, and clinical pharmacy. Through regular MDT discussions, the team can formulate a comprehensive, individualized management plan for each patient, dynamically adjusting therapeutic strategies. This collaborative effort should aim to find an optimal balance between tumor control, stabilization of blood pressure, and preservation of organ function, ultimately aiming to improve the overall prognosis of the patient.
